# Tinnitus impairs segregation of competing speech in normal-hearing listeners

**DOI:** 10.1038/s41598-020-76942-1

**Published:** 2020-11-16

**Authors:** Yang Wenyi Liu, Bing Wang, Bing Chen, John J. Galvin, Qian-Jie Fu

**Affiliations:** 1 Department of Otology and Skull Base Surgery, Eye Ear Nose and Throat Hospital, NHC Key Laboratory of Hearing Medicine, Fudan University, 83 Fenyang Road, Shanghai, 200031 China; 2 House Ear Institute, 2100 West Third Street, Los Angeles, CA 90057 USA; 3 Department of Head and Neck Surgery, David Geffen School of Medicine, University of California, Los Angeles, Los Angeles, CA 90095 USA

**Keywords:** Human behaviour, Language, Perception

## Abstract

Many tinnitus patients report difficulties understanding speech in noise or competing talkers, despite having “normal” hearing in terms of audiometric thresholds. The interference caused by tinnitus is more likely central in origin. Release from informational masking (more central in origin) produced by competing speech may further illuminate central interference due to tinnitus. In the present study, masked speech understanding was measured in normal hearing listeners with or without tinnitus. Speech recognition thresholds were measured for target speech in the presence of multi-talker babble or competing speech. For competing speech, speech recognition thresholds were measured for different cue conditions (i.e., with and without target-masker sex differences and/or with and without spatial cues). The present data suggest that tinnitus negatively affected masked speech recognition even in individuals with no measurable hearing loss. Tinnitus severity appeared to especially limit listeners’ ability to segregate competing speech using talker sex differences. The data suggest that increased informational masking via lexical interference may tax tinnitus patients’ central auditory processing resources.

## Introduction

Tinnitus can be described as perception of sound (typically noise or ringing) that is unrelated to an external stimulus. The disrupted neural activity within the auditory system has been argued to be a primary cause for tinnitus sensation^1,2^. Tinnitus of cochlear origin may affect cochlear amplification mechanisms due to deformities in cochlear function^3^. While tinnitus is often associated with hearing loss, tinnitus may also be present in individuals who exhibit normal audiometric thresholds^4^. The perceptual consequences of tinnitus in basic psychophysical measures and speech perception have been well documented in tinnitus patients with normal hearing (NH)^5–8^.

Tinnitus may affect bottom-up processing at the periphery, resulting in impaired basic auditory discrimination abilities^7–15^. Compared to NH listeners without tinnitus, some previous studies show perceptual deficits in NH listeners with tinnitus for a variety of basic auditory discrimination measures (e.g., gap detection, duration discrimination, frequency discrimination, low-frequency amplitude modulation (AM) detection, intensity discrimination)^7–14^. However, other studies have shown no differences between tinnitus and non-tinnitus listeners for other auditory measures (e.g., gap detection, intensity discrimination, high-frequency AM detection, spectral ripple discrimination, Schroeder-phase discrimination)^15–18^.

Recognition of masked speech not only requires sufficient auditory resolution for bottom-up processing, but also involves top-down (linguistic and contextual) processes. Most previous studies have shown that, compared to NH listeners without tinnitus, NH listeners with tinnitus exhibit poorer speech understanding in noise, regardless of the heterogeneity of the tinnitus population or the complexity of listening tasks^5–7,18–22^. However, the deficit in speech performance may depend on the severity of tinnitus^7^, the ear with tinnitus^18^, and task difficulty^5,22^. To varying degrees, steady noise, modulated maskers, and competing speech produce energetic, envelope, and/or informational masking^23–27^. Steady noise largely produces energetic masking at the periphery (i.e., spectral overlap between the target and masker). Modulated noise and multi-talker babble may produce energetic as well as envelope masking in cochlear regions remote from the target, and may not depend on the degree of spectral overlap between the target and the masker^28^. Competing speech may produce energetic, envelope, and informational masking (due to lexical interference, talker characteristics, etc.). Note that informational masking may occur even when there is no energetic masking, as with dichotic presentation of target and masker speech.

Segregation of competing speech may require more attention^29^ as listeners may confuse competing speech signals with the signal of interest. If top-down processes are affected by tinnitus, the ability to use different cues to segregate the target speech from intelligible competing speech would be expected to be poorer in listeners with than without tinnitus. Faraji et al.^30^ compared co-modulation release from masking between individuals with or without chronic tinnitus. While there was no significant difference in thresholds with the unmodulated masker between the tinnitus and non-tinnitus group, thresholds with the co-modulated masker were significantly higher for the tinnitus group, and co-modulation release from masking was significantly poorer for the tinnitus group. These results suggest that, compared to NH listeners without tinnitus, NH listeners with tinnitus may experience greater envelope interference and informational masking beyond the periphery.

With competing speech, the amount of informational masking has been shown to increase with the number of competing talkers, up to a certain threshold^25,31^. Previous studies have shown that informational masking may occur with two competing talkers^32–35^. For multi-talker babble, masking is reduced as the number of talkers increase beyond 2^33–35^. Previous speech perception studies in tinnitus listeners have involved steady noise^17^, multi-talker babble^7,22,36^ and 1 competing talker^17^. Kidd et al.^37^ found large masking release (MR) due to the difference in talker sex cues and/or spatial cues, which is primarily driven by the reduction in the informational masking, especially with 2-talker competing maskers. However, it is unclear whether tinnitus will affect the use of these segregation cues (talker-sex and/or spatial cues) on MR and the effects of tinnitus may differ in maskers with primarily energetic or envelope masking (e.g., steady noise or multi-talker speech babble) and in maskers with primarily informational masking (e.g., 2-talker competing speech).

In the present study, speech recognition thresholds (SRTs) were measured in NH listeners with and without tinnitus. The “tinnitus” group was comprised of individuals with normal audiometric thresholds (< 20 dB HL) and tinnitus, and the “non-tinnitus” group was comprised of individuals with normal audiometric thresholds and no tinnitus. SRTs were measured in multi-talker babble (energetic and envelope masking) and in competing speech (largely informational masking). For SRTs in competing speech, the target and masker sex were the same or different, and target and maskers were co-located or spatially separated. Similar to previous studies, SRTs were expected to be poorer in the tinnitus group than in the non-tinnitus group. Due to central processing deficits associated with tinnitus, the tinnitus group was expected to exhibit less masking release with talker sex and/or spatial segregation cues. In the tinnitus group, tinnitus severity was measured using a visual analog scale^38^ (VAS) and the Tinnitus Handicap Inventory^39^ (THI); linear regression analyses were performed between tinnitus severity and SRTs.

## Results

**Figure 1:**
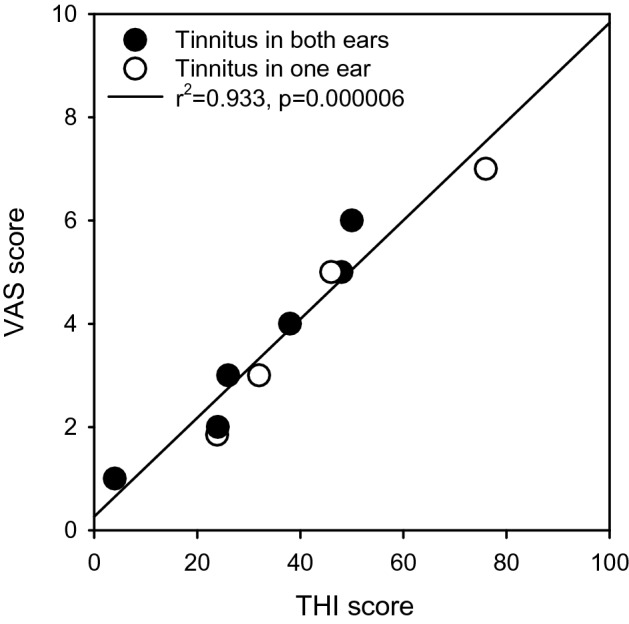
Tinnitus VAS scores as a function of THI scores. The open circles represent participants who reported tinnitus in the left or right ear only, and the filled circles represent participants who reported tinnitus in both ears. The diagonal line shows the linear regression across all data. The r^2^ and *p* values for the regression are shown at top left.

Figure 1 shows tinnitus VAS scores as a function of THI scores for the tinnitus group. For both measures, there was a wide variability in tinnitus severity, ranging from extremely mild to severe, with most participants exhibiting mild-to-moderate severity. Across all tinnitus participants, mean VAS scores were 3.7 ± 2.0 (range 1–7) and mean THI scores were 36.8 ± 19.6 (range 4–76). A student’s t-test showed no significant difference in tinnitus severity between participants who reported tinnitus in one ear (open circles; mean VAS = 3.7 ± 2.0; mean THI = 34.0 ± 18.9) or in both ears (filled circles; mean VAS = 4.0 ± 2.2; mean THI = 41.0 ± 24.1). Note that the lack of difference might also be due to the small number of participants. Linear regression analysis showed that VAS and THI scores were highly correlated (r^2^ = 0.933; *p* = 0.000006). Self-reported duration of tinnitus was also significantly correlated with VAS (r^2^ = 0.714; *p* = 0.002) and THI scores (r^2^ = 0.539; *p* = 0.008).

**Figure 2:**
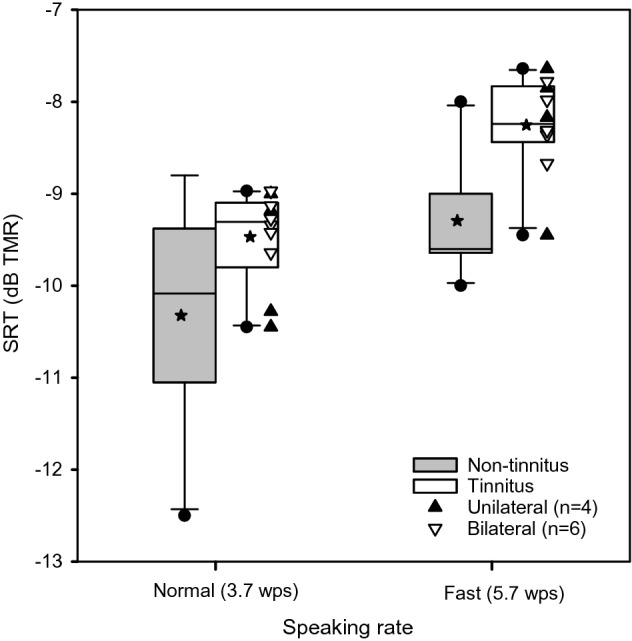
Boxplots of SRTs for MSP sentences in multi-talker babble with the normal and fast speaking rates in the non-tinnitus and tinnitus listening groups. The boxes show the 25th and 75th percentiles, the error bars show the 5th and 95th percentiles, the circles show outliers, the solid horizontal line shows the median, the stars shows the mean, and the triangles show the individual data for participants with unilateral tinnitus (filled triangle up) and bilateral tinnitus (open triangle down).

Table 1 lists mean SRTs, median SRTs and other analytics for MSP sentences in babble with the normal and fast speaking rates in the tinnitus and non-tinnitus groups. Figure 2 shows boxplots of SRTs for MSP sentences in babble with the normal and fast speaking rates in the tinnitus and non-tinnitus groups. With the normal-speaking rate, mean SRTs were − 9.47 ± 0.51 dB and − 10.32 ± 1.19 dB in the tinnitus and non-tinnitus group, respectively. With the fast speaking rate, mean SRTs were − 8.25 ± 0.52 dB and − 9.29 ± 0.63 dB in the tinnitus and non-tinnitus group, respectively. The mean difference in SRTs between the normal and fast speaking rate was comparable across groups (1.2 and 1.0 dB for the tinnitus and non-tinnitus group, respectively). The mean difference in SRTs between the tinnitus and non-tinnitus group was also comparable across speaking rates (0.9 and 1.0 dB for the normal and fast speaking rate, respectively). A Mann–Whitney non-parametric test was used to compare SRTs between subject groups (across speaking rates). Results showed that SRTs were significantly lower (better) in the non-tinnitus group than in the tinnitus group (U = 89.0, *p* = 0.003). A non-parametric Kruskal–Wallis one-way analysis of variance (ANOVA) was performed on ranked data, and post-hoc Tukey pairwise comparisons were performed between the normal and fast speaking rates within and across subject groups. Within the tinnitus group, SRTs were significantly lower with the normal rate than with the fast rate (*p* = 0.020). Within the non-tinnitus group, there was no significant difference between the normal and fast rates (*p* = 0.408). Within the normal rate, there was no significant difference between the non-tinnitus and tinnitus groups (*p* = 0.466). Within the fast rate, SRTs were significantly lower in the non-tinnitus group than in the tinnitus group (*p* = 0.027).

**Table 1:** Mean SRTs, standard deviation (Std. Dev.), confidence interval (C.I.) of the mean, maximum (max) SRT, minimum (min) SRT, median SRT, 25th and 75th percentiles for MSP sentence recognition in multi-talker babble with the normal and fast speaking rates, and CRM keyword recognition with different cue conditions, in the tinnitus and non-tinnitus groups.

Group	Test	Condition	Mean SRT	Std. Dev	C.I. of Mean	Max	Min	Median	25%	75%
Tinnitus	MSP in multi-talker babble	Normal speaking rate	− 9.5	0.5	0.4	− 9.0	− 10.5	− 9.3	− 9.8	− 9.1
		Fast speaking rate	− 8.3	0.5	0.4	− 7.6	− 9.5	− 8.2	− 8.4	− 7.8
	CMS in competing speech	No sex/no spatial	1.0	0.4	0.3	1.4	0.3	1.1	0.6	1.3
		Talker sex	− 5.1	1.5	1.1	− 2.9	− 7.1	− 4.9	− 6.6	− 3.6
		Spatial	− 5.4	0.8	0.6	− 3.6	− 6.7	− 5.5	− 6.0	− 5.1
		Talker sex + spatial	− 12.9	1.8	1.3	− 10.2	− 15.3	− 12.9	− 14.7	− 11.4
Non-tinnitus	MSP in multi-talker babble	Normal speaking rate	− 10.3	1.2	0.9	− 8.8	− 12.5	− 10.1	− 11.1	− 9.4
		Fast speaking rate	− 9.3	0.6	0.4	− 8.0	− 10.0	− 9.6	− 9.6	− 9.0
	CMS in competing speech	No sex/no spatial	− 0.7	0.7	0.5	0.1	− 2.1	− 0.6	− 1.2	− 0.1
		Talker sex	− 11.5	1.9	1.4	− 8.2	− 14.3	− 11.6	− 13.3	− 10.1
		Spatial	− 13.4	1.4	1.0	− 11.8	− 16.3	− 13.1	− 14.4	− 12.2
		Talker sex + spatial	− 17.3	1.4	1.0	− 14.3	− 18.9	− 17.3	− 18.6	− 16.3

Note that in the non-tinnitus group, 10 different participants were tested for MSP sentence recognition and for CRM keyword recognition.

**Figure 3:**
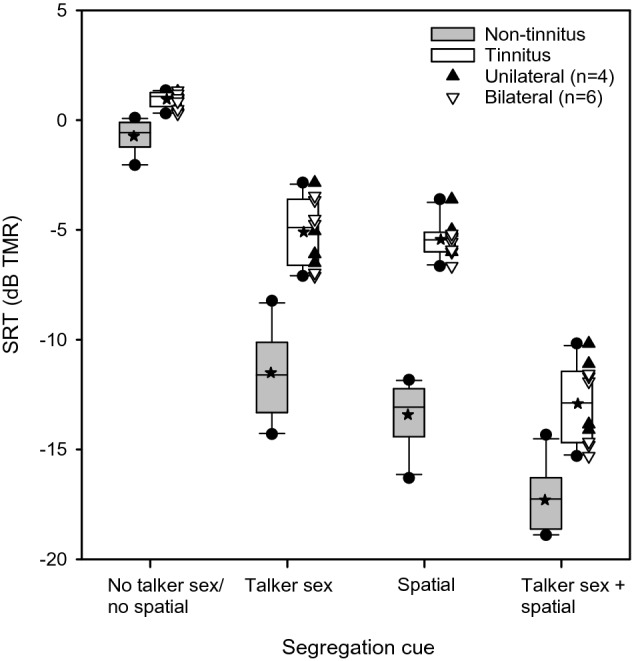
Boxplots of SRTs for competing speech for the different cue conditions in the non-tinnitus and tinnitus listener groups. The boxes show the 25th and 75th percentiles, the error bars show the 5^th^ and 95^th^ percentiles, the circles show outliers, the solid horizontal line shows the median, the stars shows the mean, and the triangles show individual data for participants with unilateral tinnitus (filled triangle up) or bilateral tinnitus (open triangle down).

Table 1 lists mean SRTs, median SRTs and other analytics for CMS sentences in competing speech for 4 cue conditions (No talker sex/no spatial, Talker sex, Spatial, Talker sex + spatial) in the tinnitus and non-tinnitus groups. Figure 3 shows boxplots of SRTs in competing speech for the 4 cue conditions in the non-tinnitus and tinnitus groups; note that participants in the non-tinnitus group were different from those in Fig. 2. In the non-tinnitus group, mean SRTs were − 0.73 ± 0.72, − 11.51 ± 1.95, − 13.41 ± 1.39, and − 17.30 ± 1.44 dB for the No talker sex/no spatial, Talker sex, Spatial, Talker sex + spatial cue conditions, respectively. In the tinnitus group, mean SRTs were 0.96 ± 0.37, − 5.09 ± 1.52, − 5.44 ± 0.82, and − 12.91 ± 1.82 dB for the No talker sex/no spatial, Talker sex, Spatial, Talker sex + spatial cue conditions, respectively. A Mann–Whitney non-parametric test was used to compare SRTs between subject groups (across cue conditions). Results showed that SRTs were significantly lower (better) in the non-tinnitus group than in the tinnitus group (U = 414.5, *p* < 0.001). A non-parametric Kruskal–Wallis one-way ANOVA was performed on ranked data, and post-hoc Tukey pairwise comparisons were performed among the cue conditions within subject groups. Within the tinnitus group, SRTs were significantly higher (poorer) in the No Sex/no spatial cue condition than in the Talker sex (*p* = 0.032), Spatial (*p* = 0.014), or Talker sex + spatial conditions (*p* < 0.001). SRTs were significantly lower (better) in the Talker sex + spatial condition than in the Talker sex (*p* = 0.014) or Spatial conditions (*p* = 0.032); there was no significant difference between the Talker sex and Spatial conditions (*p* = 0.993). Within the non-tinnitus group, SRTs were significantly higher (poorer) in the No Sex/no spatial cue condition than in the Talker sex (*p* = 0.002), Spatial (*p* < 0.001), or Talker sex + spatial conditions (*p* < 0.001). SRTs were significantly lower (better) in the Talker sex + spatial condition than in the Talker sex condition (*p* = 0.004), but not in the Spatial condition (*p* = 0.137); there was no significant difference between the Talker sex and Spatial conditions (*p* = 0.605).

**Figure 4:**
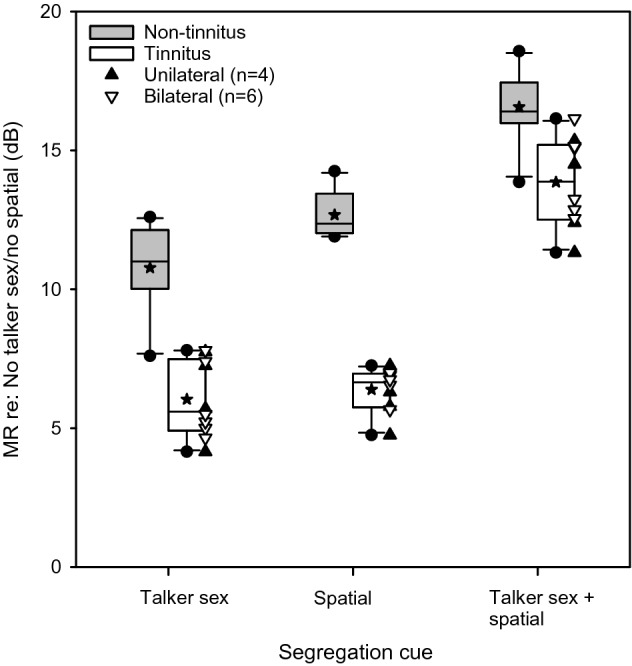
Boxplots of masking release (MR) for the different cue conditions relative to the No sex/no spatial condition in the non-tinnitus and tinnitus listener groups. The boxes show the 25th and 75th percentiles, the error bars show the 5th and 95th percentiles, the circles show outliers, the solid horizontal line shows the median, the stars shows the mean, and the triangles show individual data for participants with unilateral tinnitus (filled triangle up) or bilateral tinnitus (open triangle down).

Masking release (MR) was calculated for the Talker sex, Spatial, and Talker sex + spatial cue conditions, relative to the No talker sex/no spatial condition. Figure 4 shows boxplots of MR for the Talker sex, Spatial, and Talker sex + spatial cue conditions. In the non-tinnitus group, mean MR was 10.78 ± 1.61, 12.68 ± 0.83, and 16.57 ± 1.29 dB for the Talker sex, Spatial, and Talker sex + spatial cue conditions, respectively. In the tinnitus group, mean MR was 6.04 ± 1.37, 6.39 ± 0.78, and 13.86 ± 1.59 dB for the Talker sex, Spatial, and Talker sex + spatial cue conditions, respectively. A Mann–Whitney non-parametric test was used to compare MR between groups (across cue conditions). Results showed that MR was significantly larger in the non-tinnitus group than in the tinnitus group (U = 178.0, *p* < 0.001). A non-parametric Kruskal–Wallis one-way ANOVA was performed on ranked data, and post-hoc Tukey pairwise comparisons were performed among the cue conditions within subject groups. Within the tinnitus group, MR was significantly larger for the Talker Sex + spatial cue condition than for the Talker Sex (*p* = 0.001) or Spatial conditions (*p* = 0.005); there was no significant difference between the Talker Sex and Spatial conditions (*p* = 0.896). Within the non-tinnitus group, MR was significantly larger for the Talker Sex + spatial cue condition than for the Talker Sex (*p* < 0.001) or Spatial conditions (*p* = 0.037); there was no significant difference between the Talker Sex and Spatial conditions (*p* = 0.173).

**Figure 5:**
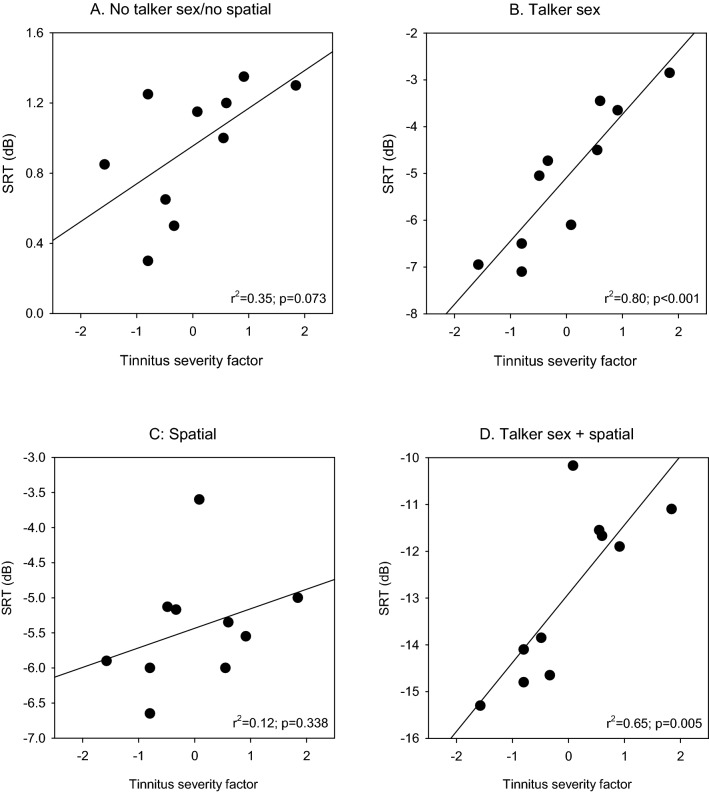
SRTs for competing speech as a function of tinnitus severity for the No talker sex/no spatial (**A**), Talker sex (**B**), Spatial (**C**), and Talker sex + spatial cue conditions (**D**). VAS and THI scores were reduced to a single “Tinnitus severity” factor using dimensionality reduction. In each panel, the diagonal line shows the linear regression fit to the data; r^2^ and *p* values are shown at the bottom right of each panel. Note that the y-axes are optimized to the SRT range for each cue condition.

Linear regression analyses were performed between tinnitus severity and SRTs in the tinnitus group. Because VAS and THI scores were highly correlated (see Fig. 1), VAS and THI data were reduced to a single “tinnitus severity” factor using dimensionality reduction. For the MSP sentences in babble, there was no significant correlation between tinnitus severity and SRTs with the normal (r^2^ = 0.07; *p* = 0.446) or fast speaking rate (r^2^ = 0.13; *p* = 0.316). Figure 5 shows SRTs for competing speech as a function of tinnitus severity for the different cue conditions. No significant correlations were observed between tinnitus severity and SRTs in the No talker sex/no spatial (panel A; r^2^ = 0.35; *p* = 0.073) or Spatial cue conditions (panel C; r^2^ = 0.12; *p* = 0.338). Significant correlations were observed between tinnitus severity and SRTs in the Talker sex (panel B; r^2^ = 0.80; *p* < 0.001) and Talker sex + spatial cue conditions (panel D; r^2^ = 0.65; *p* = 0.005); these correlations remained significant after Bonferroni correction for multiple comparisons (adjusted *p* = 0.0125). Tinnitus severity was significantly correlated with MR for the Talker sex (r^2^ = 0.69, *p* = 0.003) and Talker sex + spatial cue conditions (r^2^ = 0.62, *p* = 0.007), but not for the Spatial cue condition (r^2^ = 0.01, *p* = 0.823).

### Discussion

For all speech measures, performance was significantly poorer in the tinnitus group than in the non-tinnitus group, suggesting a general deficit regardless of test materials, masker type, or listening task, consistent with previous studies^5–7,18–22,36^. The tinnitus group was also less able to use talker sex differences and/or spatial cues to segregate competing speech, and obtained significantly less MR than did the non-tinnitus group. For the tinnitus group, tinnitus severity was highly correlated with listeners’ ability to segregate speech according to talker sex differences.

With multi-talker babble, SRTs were significantly poorer in the tinnitus group than in the non-tinnitus group; note that the mean difference in SRTs between groups was small (approximately 1.0 dB across both speaking rates). While there was no significant difference in SRTs across speaking rates in the non-tinnitus group, SRTs in the tinnitus group were significantly higher (poorer) with the fast rate than with the normal rate (mean difference = 1.2 dB). This suggests that tinnitus may have negatively affected performance as the listening demands increased. This finding is consistent with Huang et al.^19^, who measured SRTs in steady noise in NH listeners with or without tinnitus using the Mandarin Speech in Noise (MSPIN) test with high- or low-predictability sentences. They found that, for high-predictability sentences, the mean performance deficit in tinnitus group was 3.4 percentage points, relative to the non-tinnitus group. For low-predictability sentences, the mean performance deficit in tinnitus group was 12.4 percentage points, relative to the non-tinnitus group. Taken together, increased listening difficulty, whether due to lexical information (as in Huang et al.^19^) or to speaking rate (as in the present study) may better illuminate differences between tinnitus and non-tinnitus groups.

Significant and substantial deficits in SRTs in competing speech were observed in the tinnitus group, relative to the non-tinnitus group. For the No talker sex/no spatial cue condition, the mean deficit in SRTs was 1.7 dB for the tinnitus group, relative to the non-tinnitus group. However, the mean deficit in SRTs greatly increased in the tinnitus group for the Talker sex (6.4 dB), Spatial (8.0 dB), and Talker sex + spatial cue conditions (4.4 dB), relative to the non-tinnitus group. It is possible that the reduced deficit in the tinnitus group for the Talker sex + spatial condition may have been due to ceiling performance effects in the non-tinnitus group, where the mean SRT was − 17.3 dB. Different from the pattern of results for SRTs in babble, SRTs in competing speech were most similar between the tinnitus and non-tinnitus groups for the most challenging listening condition (No talker sex/no spatial), and diverged across groups as segregation cues became available. As such, the tinnitus group was less able to utilize segregation cues than was the non-tinnitus group. For competing speech with no talker sex or spatial cues, the degree of masking may have been sufficiently high to obscure differences between the tinnitus and non-tinnitus groups.

Although SRTs with competing speech were significantly lower (better) for the non-tinnitus group than for the tinnitus group, utilization of segregation cues was similar across groups (Fig. 3). In both groups, SRTs were significantly lower for the Talker sex, Spatial, and Talker sex + spatial cue conditions, relative to the No talker sex/no spatial condition. In both groups, there was no significant difference between the very different Talker sex and Spatial cue conditions. While the tinnitus and non-tinnitus groups may have similarly utilized segregation cues, utilization efficiency was significantly poorer in the tinnitus group than in the non-tinnitus group. This is reflected by the significantly greater MR in the non-tinnitus group than in the tinnitus group (Fig. 4).

The present findings are not consistent with Zeng et al.^17^, who found no significant difference in utilization of talker sex cues to segregate competing speech between tinnitus and non-tinnitus listeners. Differences in test materials and methods may partly explain discrepancies between studies. A closed-set CRM task using Mandarin matrix-styled sentences was used in the present study, while an open-set sentence recognition task using low-predictability English sentences was used in Zeng et al.^17^. Two competing speech maskers were used in present study, while three different masker conditions were tested in Zeng et al.^17^ (i.e., steady noise, 1 competing female talker, or 1 competing male talker). Previous studies have shown that the amount of informational masking increases as the number of competing talkers increases from 1 to 2^24,25^. Masker sentences were randomly generated for each test trial in the present study; a single masker sentence appears to have been used for the competing female or male masker in Zeng et al.^17^, which might have allowed some entrainment to the masker sentence across test trials and runs, effectively reducing informational masking. Taken together, the present study methods and materials may have generally increased informational masking, allowing for better differentiation between tinnitus and non-tinnitus listeners than observed in Zeng et al.^17^.

The present data are in general agreement with most previous studies that show masked speech perception is poorer in individuals with tinnitus than those without tinnitus ^5–7,18–22,36^. Relatively few studies have reported correlations between tinnitus severity and masked speech perception. In the present study, tinnitus severity was not significantly correlated with MSP sentence recognition in multi-talker babble, with the normal or fast speaking rate. This is consistent with Jain and Sahoo^7^, who showed no significant correlation between tinnitus severity and speech understanding in 4-talker speech babble. The present data also showed no significant correlation between tinnitus severity and SRTs with two co-located male talkers (Fig. 5A) or two spatially separated male-talkers (Fig. 5C). However, there was a significant correlation between tinnitus severity and SRTs with two co-located female talkers (Fig. 5B) or two spatially separated female talkers (Fig. 5D). According to Kidd et al.^37^, talker sex differences allow for a release from informational masking. The addition of spatial cues may enhance talker sex cues, allowing for greater release from informational masking^40,41^. It is unclear why tinnitus severity was unrelated to utilization of spatial cues to segregate competing speech in the present study. It is possible that head shadow effects and improved TMR at each ear (relative to co-located speech and maskers) may not be influenced by tinnitus, as these more represent the physical aspects of the target and maskers.

Besides the present correlation between tinnitus severity and segregation of competing speech according to talker sex differences, tinnitus severity has been negatively correlated with other measures, such as the ability to control the emotional response to tinnitus^42^ and quality of life^43^. Thompson et al.^43^ also found that higher levels of physical activity (e.g., exercise) were correlated with lower tinnitus severity scores, possibly due to stress reduction. Using functional magnetic resonance imaging (fMRI), Carpenter-Thompson et al.^44^ found that tinnitus severity was associated with activation of frontal areas, with lower severity associated with greater frontal activation. The authors suggested that individuals with lower tinnitus severity may better utilize frontal regions to better control their emotional response to the affective sounds. Voice pitch is the most important cue for voice emotion recognition and voice gender discrimination^45^. It is possible that individuals with lower tinnitus severity may better utilize frontal regions to segregate competing speech according to talker sex differences.

Deficits in central auditory processing and/or cognitive function may significantly affect masked speech understanding^32,46,47^. Ivansic et al.^21^ suggested that difficulties in understanding speech in noise in tinnitus patients may be due to deficits in central processing and/or attention, and further suggested that more complex listening tasks may better reveal central difficulties for tinnitus patients. Tegg-Quinn et al.^48^ suggested that tinnitus patients may have difficulties in allocating attention resources, which may affect cognitive processes needed to segregate speech from noise or from competing speech. Overall, the present data highlight the potential role of central processing deficits on speech performance in tinnitus patients, compared to non-tinnitus listeners. Tinnitus severity was not correlated with SRTs with speech babble, but was significantly correlated with SRTs for competing speech when the target and masker sex was different, and when talker sex differences were combined with spatial cues. However, MR due to talker sex differences (i.e., release from informational masking, which is more central in origin) was smaller in the tinnitus group than in the non-tinnitus group (Fig. 4). The present data suggest that segregation of target and multiple masker sentences (largely, release from informational masking) may better reveal central processing difficulties associated with tinnitus than when measuring speech understanding in multi-talker babble (which may more represent release from envelope masking or from some combination of energetic and informational masking).

One potential limitation of the present study is the relatively small number of participants in the tinnitus group. The mean performance difference was often quite large between tinnitus and non-tinnitus group, especially for segregation of competing speech (Fig. 3). Age effects on speech performance were somewhat controlled by including individuals with similar ranges across the listener groups; also, the maximum age was 45 years, which may be sufficiently low to avoid large age effects. However, the characteristics and distribution of tinnitus may be heterogeneous. Testing with a large group of participants with various characteristics of tinnitus may result in a different pattern of results. The limited data from the present study showed that there was no significant difference in performance between participants with bilateral or unilateral tinnitus. However, etiology, duration, and sound quality of tinnitus may also affect performance in tinnitus patients. The present number of tinnitus participants was too small to explore the potential roles of these factors on understanding of masked speech. Further studies with more participants may provide additional information about underlying mechanisms of tinnitus that may limit understanding of masked speech.

Another potential limitation was the use of a non-individualized head related transfer function (HRTF) to measure segregation of competing speech. Using an HRTF that is not listener-specific may result in an unrealistic perception of space or insufficient externalization of sound, which may especially affect SRTs with spatial cues. In previous related studies, segregation of competing speech was measured using a non-individualized HRTF^32^ or loudspeakers^49^ using similar methods and the same cue conditions used in the present study. In Zhang et al.^32^, mean SRTs with the non-individualized HRTF were 0.36 ± 1.95, − 8.31 ± 1.81, − 11.87 ± 2.79, − 12.62 ± 2.69 for the No talker sex/no spatial, Talker sex, Spatial, Talker sex + spatial cue conditions, respectively. In Willis et al.^49^, mean SRTs with loudspeakers were 1.23 ± 1.28, − 7.77 ± 2.45, − 11.65 ± 3.10, − 12.01 ± 2.54 dB for the No talker sex/no spatial, Talker sex, Spatial, Talker sex + spatial cue conditions, respectively. SRTs data were comparable across these studies, despite difference in sound presentation. These data suggest that while using a non-individualized HRTF in headphones may not be ideal, that pattern of results were similar as with using real loudspeakers. As such, using a non-individualized HRTF was not likely to be a limiting factor for perception of competing speech in the present study.

## Materials and methods

In compliance with ethical standards for human subjects, written informed consent was obtained from all participants or their legal guardians before proceeding with any of the study procedures. The study and its consent procedure were approved by the local ethics committee (Ethics Committee of Eye and Ear, Nose, Throat Hospital of Fudan University; approval number: KY2012-009). All research was performed in accordance with relevant guidelines and regulations.

### Participants

Thirty Mandarin-speaking Chinese NH listeners were recruited for this study. Ten were clinically diagnosed as having tinnitus (6 males and 4 females; mean age at testing = 27.9 ± 9.1 yrs, range = 15–43 yrs), and 20 had no diagnosis or self-report of tinnitus (10 males and 10 females; mean age at testing = 27.8 ± 6.5 yrs, range = 22–45 yrs). All participants had pure tone thresholds < 25 dB HL at all audiometric frequencies between 250 and 8000 Hz measured using pure-tone audiometry administered in a sound treated room. Within the tinnitus group, tinnitus originated from the right ear in 1 participant, from the left ear in 3 participants, and from both ears in 6 participants. Table 2 shows the demographic information for the 10 tinnitus participants.

**Table 2:** Demographic information for the tinnitus group.

Participant	Gender	Age (years)	VAS	THI	Tinnitus ear	Tinnitus duration (years)
S1	F	37	4	38	Right	0.33
S2	M	43	3	26	Left	0.25
S3	F	26	2	24	Left	0.17
S4	M	18	5	48	Both	5.00
S5	M	21	3	32	Both	0.17
S6	F	20	5	46	Both	4.00
S7	M	15	1	4	Both	0.17
S8	M	35	7	76	Left	4.00
S9	M	34	6	50	Both	4.00
S10	F	30	2	24	Both (L > R)	0.33

*VAS* visual analog scale score for tinnitus severity, *THI* tinnitus handicap inventory score.

### Tinnitus severity measures

Tinnitus severity was measured using a visual analog scale^38^ (VAS) and the Tinnitus Handicap Inventory^39^ (THI). For VAS measures, participants were asked to indicate their current tinnitus severity by marking the number (0–10) on a 10-cm line (which included 1-cm ticks and corresponding 0–10 number) that was anchored with the extreme labels “No tinnitus at all” and “Worst tinnitus imaginable.” The THI is a validated subjective, self-reported rating of the impact of tinnitus on patients’ everyday life. The THI contains 25 questions; listeners respond with Yes (4 points), Sometimes (2 points, or No (0 points), resulting in a possible maximum score of 100. A THI score of 0–16 indicates "no or slight handicap", 18–36 indicates "mild" handicap, 38–56 indicates "moderate" handicap, 58–76 indicates "severe" handicap, and a score of 78–100 indicates "catastrophic handicap".

### Test stimuli and procedure

SRTs for sentence recognition in six-talker babble was measured using the Mandarin Speech Perception (MSP) test materials^50^, which consists of 5 lists of 20 sentences each. Each sentence contains 7 monosyllabic words, resulting in a total of 140 monosyllabic words for each list. The MSP materials consist of high-quality digital recordings of speech produced by a female Mandarin talker at two speaking rates (normal: 3.7 words per second; fast: 5.7 words per second). SRTs were measured using an open-set test paradigm and an adaptive procedure that converged on target-to-masker ratio (TMR) that produced 50% correct word-in-sentence recognition. One of five MSP lists was randomly selected for testing at each speaking rate. Stimuli were delivered via Sennheiser HDA300 headphones connected to the headphone output of a clinical audiometer, which was connected to an external audio device (Edirol UA-25EX). The audio device was connected to a Windows 10 computer via USB. Participants were seated in a sound-treated audio booth during testing. The target and masker were presented diotically (i.e., from both the right and left channels). The target sentence was always presented at 65 dBA (calibrated via clinical audiometer), and the masker level was adjusted according to the correctness of response. Custom software (iSTAR™; http://istar.emilyfufoundation.org) was used to administer the test, calculate the TMR during testing, and calculate the SRT at the end of the test run. During each test trial, the TMR was calculated according to the long-term root mean square (RMS) amplitude of the target sentence and the masker. During testing, a sentence was randomly selected from the list. Participants were instructed to repeat the sentence as accurately as possible, and to guess if they were unsure. The experimenter clicked on the correctly identified words, and the software calculated the percent of words correctly identified. If the listener repeated 50% or more words correctly, the TMR was reduced by 2 dB; if not, the TMR was increased by 2 dB. A reversal occurred when the change in the TMR switched from decreasing to increasing or vice versa. Each test run (20 trials) typically had 6 to 10 reversals in TMR. The SRT for each test run was calculated by averaging the TMR across the last 6 reversals.

The Closed-set Mandarin Speech (CMS) corpus^51,52^ was used to measure segregation of competing speech. The CMS corpus consists of matrix-styled test materials which can be used to randomly create five-word sentences with same grammatical structure: name, verb, number, color, and object, each of which contain 10 words. The CMS materials were used to generate target and masker sentences. The target sentences were produced by a male talker; the mean fundamental frequency (F0) across all words was 139 Hz. Masker sentences were produced by 2 males (mean F0s = 143 Hz and 178 Hz) or 2 females (mean F0s = 208 Hz and 248 Hz). A coordinate response matrix (CRM) test paradigm was used, similar to previous studies^24,47,49,52^. Listeners were asked to identify keywords from the Number and Color categories that were embedded in the randomly generated five-word sentences. Here, SRTs were defined as the TMR that produced 100% correct keyword recognition, consistent with the CRM test paradigm. The first word in the target sentence was always the Name “Xiaowang,” followed by randomly selected words from the remaining four categories. Two masker sentences produced by the male or female talkers were randomly generated using words not contained in the target sentence and different across masker sentences. An example target sentence could be “Xiaowang sold Three Red strawberries,” while the masker sentences could be the combination “Xiaozhang saw Two Blue kumquats” and “Xiaodeng took Eight Green papayas.” Target and masker sentences were individually processed using the HRTF from Willis et al.^49^ to simulate various spatial locations. SRTs were measured for 4 cue conditions: (1) No talker sex/no spatial (male target and male maskers, all originating from 0°), (2) Talker sex (male target and female maskers, all originating from 0°), (3) Spatial (male target originating from 0°, male maskers originating from 90° and 270°), and (4) Talker sex + spatial (male target originating from 0°, female maskers originating from 90° and 270°). Stimuli were delivered via Sennheiser HDA300 headphones connected to the headphone output of clinical audiometer, which was connected to an external audio device (Edirol UA-25EX). The audio device was connected to a Windows 10 computer via USB. The target sentence was always presented at 65 dBA. Participants were seated in a sound-treated audio booth during testing. The presentation level of the masker sentences was adjusted according to the TMR in each trial. For example, for a 10 dB TMR, the masker sentences were presented at 55 dBA. If the listener correctly identified both keywords, the TMR was reduced by 2 dB; if not, the TMR was increased by 2 dB. The SRT was calculated by averaging the last 6 reversals in TMR. Three test runs were completed for each listening condition and the SRT was averaged across runs. The four conditions were randomized within and across participants. Custom software (Angel Sound™; http://angelsound.emilyfufoundation.org) was used to calculate the TMR and SRT during testing.

### Data analysis

Speech recognition in multi-talker babble and competing talkers was analyzed in terms of SRT. Masking release (MR) was calculated for the Talker sex, Spatial, and Talker sex + spatial cue conditions, relative to the No talker sex/no spatial condition. SRT data were analyzed using non-parametric tests (Mann–Whitney test to compare listener groups; Kruskal–Wallis one-way ANOVAs on ranked data with post-hoc Tukey pairwise comparisons to compare test conditions). VAS and THI data were reduced to a single “tinnitus severity” factor using dimensionality reduction. Linear regression analyses were performed to determine relationships between tinnitus severity and SRTs. Analyses were performed using Systat software (v. 14) or SPSS (v. 22). For most analyses, the significance level was *p* = 0.05; for pairwise comparisons, the significance level was adjusted to control for multiple comparisons using Tukey or Bonferroni corrections.

## Data Availability

The data used for the current study are provided as supplementary material.
